# Correction: Cyclin B1 protects against meniscal extracellular matrix catabolism and degeneration via the NOD-like receptor pathway

**DOI:** 10.1042/CS20260446_COR

**Published:** 2026-10-14

**Authors:** Hong Guan, Jiangwen Cheng, Xinghong Yuan, Daozhang Cai, Haiyan Zhang, Liangliang Liu

**Affiliations:** 1 Department of Joint Surgery, Center for Orthopedic Surgery, The Third Affiliated Hospital, Southern Medical University, Guangzhou, China; 2 Department of Orthopedics, Orthopedic Hospital of Guangdong Province, Academy of Orthopedics, Guangdong Province, The Third Affiliated Hospital, Southern Medical University, Guangzhou, China; 3 The Third School of Clinical Medicine, Southern Medical University, Guangzhou, China; 4 Guangdong Provincial Key Laboratory of Bone and Joint Degeneration Diseases, Guangzhou, China

**Keywords:** CCNB1, meniscal degeneration, NLR-related protein 3, Osteoarthritis

The authors of the original article “Cyclin B1 protects against meniscal extracellular matrix catabolism and degeneration via the NOD-like receptor pathway” (DOI: 10.1042/CS20260446) would like to correct the author contribution list and confirm that the authors listed first (Hong Guan), second (Jiangwen Cheng) and third (Xinghong Yuan) contributed equally to this work as first co-authors.

The requested correction has been assessed and approved by the Editorial Board.

The authors declare that these corrections do not change the results or conclusions of their paper.

